# Supplementary material for: Resolving the full spectrum of human genome variation using Linked-Reads
Source: Genome Res. 2019 Apr;29(4):635–45. doi: 10.1101/gr.234443.118 (PMC6442396; doi:10.1101/gr.234443.118)
Supplement: Supplemental Material [file supp_gr.234443.118_Supplemental_longranger-2.2.zip › longranger-2.2.tar.bz2!/lr2.2-public/lib/stan/cmdstan/stan/doc/api/html/agrad_2rev_2matrix_2multiply__lower__tri__self__transpose_8hpp_source.html]

Stan: src/stan/agrad/rev/matrix/multiply\_lower\_tri\_self\_transpose.hpp Source File


|  |  |
| --- | --- |
|  | Stan  2.5.0  probability, sampling & optimization |


- Main Page
- Related Pages
- Namespaces
- Classes
- Files

- File List
- File Members

All Classes Namespaces Files Functions Variables Typedefs Enumerations Enumerator Friends Macros Pages

- src
- stan
- agrad
- rev
- matrix

multiply\_lower\_tri\_self\_transpose.hpp

Go to the documentation of this file.

1 #ifndef STAN\_\_AGRAD\_\_REV\_\_MATRIX\_\_MULTIPLY\_LOWER\_TRI\_SELF\_TRANSPOSE\_HPP

2 #define STAN\_\_AGRAD\_\_REV\_\_MATRIX\_\_MULTIPLY\_LOWER\_TRI\_SELF\_TRANSPOSE\_HPP

3

4 #include <vector>

5 #include <boost/math/tools/promotion.hpp>

6 #include <stan/math/matrix/Eigen.hpp>

7 #include <stan/math/matrix/typedefs.hpp>

8 #include <stan/math/error\_handling/matrix/check\_square.hpp>

9 #include <stan/agrad/rev/var.hpp>

10 #include <stan/agrad/rev/numeric\_limits.hpp>

11 #include <stan/agrad/rev/matrix/typedefs.hpp>

12 #include <stan/agrad/rev/matrix/dot\_product.hpp>

13 #include <stan/agrad/rev/matrix/dot\_self.hpp>

14

15 namespace stan {

16  namespace agrad {

17

18  inline matrix\_v

19  multiply\_lower\_tri\_self\_transpose(const matrix\_v& L) {

20  //stan::math::check\_square("multiply\_lower\_tri\_self\_transpose(%1%)",

21  //L,"L",(double\*)0);

22  int K = L.rows();

23  int J = L.cols();

24  matrix\_v LLt(K,K);

25  if (K == 0) return LLt;

26  // if (K == 1) {

27  // LLt(0,0) = L(0,0) \* L(0,0);

28  // return LLt;

29  // }

30  int Knz;

31  if (K >= J)

32  Knz = (K-J)\*J + (J \* (J + 1)) / 2;

33  else // if (K < J)

34  Knz = (K \* (K + 1)) / 2;

35  vari\*\* vs = (vari\*\*)memalloc\_.alloc( Knz \* sizeof(vari\*) );

36  int pos = 0;

37  for (int m = 0; m < K; ++m)

38  for (int n = 0; n < ((J < (m+1))?J:(m+1)); ++n) {

39  vs[pos++] = L(m,n).vi\_;

40  }

41  for (int m = 0, mpos=0; m < K; ++m, mpos += (J < m)?J:m) {

42  LLt(m,m) = var(new dot\_self\_vari(vs + mpos, (J < (m+1))?J:(m+1)));

43  for (int n = 0, npos = 0; n < m; ++n, npos += (J < n)?J:n) {

44  LLt(m,n) = LLt(n,m) = var(new dot\_product\_vari<var,var>(vs + mpos, vs + npos, (J < (n+1))?J:(n+1)));

45  }

46  }

47  return LLt;

48  }

49

50  }

51 }

52 #endif

typedefs.hpp

typedefs.hpp

stan::agrad::memalloc\_

memory::stack\_alloc memalloc\_

**Definition:** var\_stack.cpp:16

dot\_self.hpp

dot\_product.hpp

stan::agrad::matrix\_v

Eigen::Matrix< var, Eigen::Dynamic, Eigen::Dynamic > matrix\_v

The type of a matrix holding stan::agrad::var values.

**Definition:** typedefs.hpp:21

stan::agrad::vari

The variable implementation base class.

**Definition:** vari.hpp:28

var.hpp

Eigen.hpp

stan::memory::stack\_alloc::alloc

void \* alloc(size\_t len)

Return a newly allocated block of memory of the appropriate size managed by the stack allocator...

**Definition:** stack\_alloc.hpp:168

stan::agrad::var

Independent (input) and dependent (output) variables for gradients.

**Definition:** var.hpp:27

stan::agrad::multiply\_lower\_tri\_self\_transpose

Eigen::Matrix< fvar< T >, R, R > multiply\_lower\_tri\_self\_transpose(const Eigen::Matrix< fvar< T >, R, C > &m)

**Definition:** multiply\_lower\_tri\_self\_transpose.hpp:17

numeric\_limits.hpp

check\_square.hpp


---

[ Stan Home Page ]

*© 2011–2014,
Stan Development Team.*
